# The effect of menaquinone-7 supplementation on vascular calcification in patients with diabetes: a randomized, double-blind, placebo-controlled trial

**DOI:** 10.1093/ajcn/nqz147

**Published:** 2019-08-06

**Authors:** S R Zwakenberg, P A de Jong, J W Bartstra, R van Asperen, J Westerink, H de Valk, R H J A Slart, G Luurtsema, J M Wolterink, G J de Borst, J A van Herwaarden, M A van de Ree, L J Schurgers, Y T van der Schouw, J W J Beulens

**Affiliations:** 1 Julius Center for Health Sciences and Primary Care, University Medical Center Utrecht, Utrecht University, Utrecht, Netherlands; 2 Department of Radiology, University Medical Center Utrecht, Utrecht University, Utrecht, Netherlands; 3 Department of Vascular Medicine, University Medical Center Utrecht, Utrecht University, Utrecht, Netherlands; 4 Department of Endocrinology, University Medical Center Utrecht, Utrecht University, Utrecht, Netherlands; 5 Medical Imaging Center, Department of Nuclear Medicine and Molecular Imaging, University of Groningen, University Medical Center Groningen, Groningen, Netherlands; 6 Image Sciences Institute, University Medical Center Utrecht, Utrecht University, Utrecht, Netherlands; 7 Department of Vascular Surgery, University Medical Center Utrecht, Utrecht University, Utrecht, Netherlands; 8 Department of Internal Medicine, Diakonessenhuis, Utrecht, Netherlands; 9 Department of Biochemistry, Cardiovascular Research Institute Maastricht, Maastricht University, Maastricht, Netherlands; 10 Department of Epidemiology & Biostatistics, Amsterdam Public Health Research Institute, Amsterdam University Medical Centers, Amsterdam, Netherlands

**Keywords:** menaquinone-7, vitamin K, vascular calcification, cardiovascular disease, diabetes

## Abstract

**Background:**

Vitamin K occurs in the diet as phylloquinone and menaquinones. Observational studies have shown that both phylloquinone and menaquinone intake might reduce cardiovascular disease (CVD) risk. However, the effect of vitamin K on vascular calcification is unknown.

**Objectives:**

The aim of this study was to assess if menaquinone supplementation, compared to placebo, decreases vascular calcification in people with type 2 diabetes and known CVD.

**Methods:**

In this double-blind, randomized, placebo-controlled trial, we randomly assigned men and women with type 2 diabetes and CVD to 360 µg/d menaquinone-7 (MK-7) or placebo for 6 mo. Femoral arterial calcification at baseline and 6 mo was measured with ^18^sodium fluoride positron emission tomography (^18^F-NaF PET) scans as target-to-background ratios (TBRs), a promising technique to detect active calcification. Calcification mass on conventional computed tomography (CT) scan was measured as secondary outcome. Dephosphorylated–uncarboxylated matrix Gla protein (dp-ucMGP) concentrations were measured to assess compliance. Linear regression analyses were performed with either TBR or CT calcification at follow-up as the dependent variable, and treatment and baseline TBR or CT calcification as independent variables.

**Results:**

We randomly assigned 35 patients to the MK-7 group (33 completed follow-up) and 33 to the placebo group (27 completed follow-up). After the 6-mo intervention, TBR tended to increase in the MK-7 group compared with placebo (0.25; 95% CI: −0.02, 0.51; *P* = 0.06), although this was not significant. Log-transformed CT calcification mass did not increase in the intervention group compared with placebo (0.50; 95% CI: −0.23, 1.36; *P* = 0.18). MK-7 supplementation significantly reduced dp-ucMGP compared with placebo (−205.6 pmol/L; 95% CI: −255.8, −155.3 pmol/L). No adverse events were reported.

**Conclusion:**

MK-7 supplementation tended to increase active calcification measured with ^18^F-NaF PET activity compared with placebo, but no effect was found on conventional CT. Additional research investigating the interpretation of ^18^F-NaF PET activity is necessary. This trial was registered at clinicaltrials.gov as NCT02839044.

## Introduction

Vascular calcification is associated with a 3- to 4-fold increased risk of cardiovascular events (1, 2). In the past, vascular calcification was viewed as a passive process of calcium deposition, but evidence has shown that vascular calcification is an active process regulated by stimulators and inhibitors (3). Matrix Gla protein (MGP), a vitamin K-dependent protein, functions as an inhibitor of vascular calcification (4). This function of MGP was first shown in MGP knockout mice, which resulted in initiation and progression of vascular calcification (4). Multiple observational studies have since shown that low levels of inactive MGP, dephosphorylated–uncarboxylated MGP (dp-ucMGP), are associated with less vascular calcification and reduced cardiovascular disease (CVD) risk (5). Vitamin K is suggested to reduce vascular calcification and risk of CVD.

Vitamin K is fat-soluble and occurs in 2 different forms, phylloquinone (vitamin K-1) and menaquinones (vitamin K-2). Phylloquinone is mainly derived from green leafy vegetables, whereas menaquinones mainly occur in fermented animal products such as cheese and meat (6). Phylloquinone and menaquinones have the same chemical structure but differ in the length and saturation degree of the side chain (7). Menaquinones have a longer half-life and a higher bioavailability.

Multiple observational studies have shown a reduced CVD risk with high vitamin K intake (5), although the evidence is not conclusive. To date, 2 studies have investigated the effect of phylloquinone supplementation on vascular calcification and have shown reduced progression in older men and women (8) and in chronic kidney disease patients (9). The effect of menaquinones on vascular calcification has not been investigated, although 2 intervention studies have shown a reduced vascular stiffness after menaquinone supplementation (10, 11), and another study did not find an effect of menaquinone supplementation on vascular stiffness or other markers of vascular health (12). None of the trials investigated the effect of vitamin K and vascular calcifications in people with type 2 diabetes, although vascular calcification is prevalent in ∼70% of people with type 2 diabetes (1). Finally, vascular calcification is an active process, but none of the studies measured the active vascular calcification. ^18^Sodium fluoride positron emission tomography (^18^F-NaF PET) is a promising technique to detect early changes in active vascular calcification before calcifications become visible by computed tomography (CT) (13). Therefore, this double-blind, randomized placebo-controlled trial aimed to investigate the effect of 6 mo of menaquinone-7 (MK-7) supplementation on active vascular calcification, measured by ^18^F-NaF PET activity, in people with type 2 diabetes and a history of CVD.

## Methods

### Study design and study population

This study is a double-blind, randomized placebo-controlled trial. Participants were recruited through a pre-existing diabetes cohort study (Utrecht participants only) (14), a Julius Center database of subjects who are interested in participating in studies, and via outpatient clinics of the University Medical Center Utrecht and Diakonessenhuis Utrecht. Participants who met the following criteria were included in the study: men and women aged >40 y with diagnosed type 2 diabetes and pre-existing CVD, because vascular calcification is highly prevalent in this patient group, and an estimated glomerular filtration rate (eGFR) >30. Exclusion criteria were vitamin K antagonist use, use of (multi)vitamins with vitamin K, unwillingness to stop vitamin K use before randomization, and known coagulation problems such as deep vein thrombosis. All participants gave written informed consent prior to participation. This trial was approved by the institutional review board of the University Medical Center Utrecht and registered at clinicaltrials.gov as as NCT02839044.

### Intervention

Participants were randomly assigned to 360 µg MK-7 daily or a placebo supplement for 6 mo. Participants received daily 2 tablets of MK-7 (Nattopharma) or placebo (Legosan), which were similar in taste and appearance. Participants were asked to take the tablets with their evening meal. At the start and at the end of the study, MK-7 and placebo tablets were measured for MK-7 content to assess the stability of the tablets (Maastricht University). HPLC, reversed-phase C-18 column, and fluorometric after post column electrochemical reduction were used to determine the free vitamin K-2 (MK-7) concentration. During the entire study, the MK-7 content was stable, with 262 µg MK-7 at the start and 223 µg MK-7 at the end of the study. Because this is not a standard biomarker test, this might be an overestimation. Participants returned their leftover tablets, and we calculated the compliance as the number of tablets actually taken divided by the number of tablets that should have been taken. dp-ucMGP serves as a marker for vitamin K status, where low dp-ucMGP concentrations represent long-term high vitamin K intake (15). Therefore, dp-ucMGP was measured at baseline, 3 mo, and 6 mo to assess whether participants were compliant to the treatment. dp-ucMGP was measured with the sandwich ELISA method using the IDS Automated Analyser IDS-iSYS InaKtif MGP assay (Maastricht University).

Participants were randomly assigned in a 1:1 ratio, stratified for sex, performed by the data management department through a computerized method. A list of numbers was created, and the researcher only received a medication number. During the study, the randomization code was kept at the data management and pharmacy departments.

### Study endpoints

The primary outcome was active vascular calcification, measured as femoral arterial wall ^18^F-NaF PET activity measured at baseline and 6 mo. ^18^F-NaF PET activity is promising for detecting early changes in active deposition of vascular calcification before calcifications become visible on CT, due to the binding of the tracer to hydroxyapatite, thereby enabling the detection of microcalcifications. Therefore, this method is suitable to detect changes in active calcification during a 6-mo intervention period. ^18^F-NaF PET activity is prevalent in the femoral arteries because medial calcification mostly resembles bone formation. In addition, femoral uptake can easily be evaluated without activity spillover from adjacent bones, in contrast to abdominal aortic uptake (16).

^18^F-NaF PET CT scans were conducted at baseline and after 6 mo on a Siemens Biograph 40 scanner (Siemens Healthcare). Ninety minutes before imaging, participants received an intravenous injection of 2.0 MBq/kg ^18^F-NaF, with a maximum dosage of 200 MBq. The primary outcome was the target-to-background ratio (TBR) measured in the femoral artery.

The maximal standardized uptake value (SUV_max_) was measured in the left and right femoral artery, from the bifurcation of the femoral artery to the femur condyles (IntelliSpace Portal v8.0; Philips Healthcare). Slice thickness was 5 mm, with a slice interval of 4 mm. The mean of the SUV_max_ of the left and right femoral artery was calculated (mean number of 47 measurements per patient). Second, the blood pool was determined in the superior vena cava on consecutive slices starting at the aortic arch. Three measurements of the mean standardized uptake value (SUV_mean_) were performed and an average was used. The femoral TBR was calculated by the SUV_max_ in the femoral artery divided by the SUV_mean_ in the vena cava. Vascular calcification on conventional CT images was measured as a secondary outcome. Calcification in both left and right femoral artery was measured using in-house–developed software (iX Viewer; Image Sciences Institute), using a threshold of 130 Hounsfield units for calcium. Calcification mass score was computed as the product of the volume of the lesion (in milliliters) and the mean attenuation (in Hounsfield units) of the lesion (17). Similar to the PET/CT images, we measured calcification from the bifurcation of the femoral artery to the femur condyles. Both TBR and arterial calcification measurements were performed by 1 investigator (RvA), blinded for treatment and patient characteristics. Three investigators, blinded for all characteristics, assessed 10 randomly selected participants (5 baseline and 5 follow-up measurements) to calculate the interobserver reliability for TBR measurements, and 20 randomly selected participants for calcification mass (10 baseline and 10 follow-up measurements). The intraclass correlation for the interobserver reliability was 0.98 (95% CI: 0.94, 0.99) for TBR measurements and 0.998 (95% CI: 0.995, 0.999) for calcification mass using conventional CT imaging.

### Other measurements

Participants visited the University Medical Center Utrecht at baseline and after 3 and 6 mo. During these visits, lifestyle questionnaires were obtained, including disease history, medication use, and smoking habits. During each visit, anthropometric measurements including height, weight, and waist and hip circumference were performed twice, and a mean value was calculated. Ankle–brachial index (ABI), a measurement of arterial stiffness, was measured at baseline. ABIs were calculated by dividing the highest average arm systolic blood pressure by the highest of the ankle pressures of that leg. Blood pressure was measured twice in the sitting position using an automated oscillomat (Omron HEM-907). Nonfasting blood samples were drawn, and glycated hemoglobin, creatinine, and lipid levels were measured. eGFR was calculated using the MDRD formula. Finally, participants were asked to complete a 3-d food record to obtain the dietary vitamin K intake. The food record included 3 nonconsecutive days, including 2 weekdays and 1 weekend day, and consisted of a prespecified format for breakfast, lunch, dinner, and snacks. The food records were analyzed using Evry (Ensemble BV), based on the Dutch national food composition table (2013) (18) and a previously described vitamin K food content database (19). The energy-adjusted intakes of total vitamin K, phylloquinone, and menaquinones were calculated using the residual method.

### Statistical analysis

The sample size was calculated based on a previous study including ^18^F-NaF PET CT, showing a mean TBR of 1.96 (16). With a power of 80%, a 2-sided α of 5%, an SD of the difference in TBR between baseline and follow-up of 0.41, and a 15% dropout rate, 70 participants were required to detect a 15% difference in TBR. A 15% difference was also found in previous studies investigating the effect of vitamin K on coronary artery calcification (8, 9).

Baseline characteristics were described as percentages, means ± SDs, or medians (IQRs) as appropriate per treatment arm, and clinically relevant baseline differences between treatment arms were visually assessed. The primary endpoint was TBR, and log-transformed calcification mass was included as a secondary endpoint. Absolute differences between baseline and follow-up were calculated. Linear regression analyses were performed with the follow-up calcification measurement as outcome and treatment and baseline calcification measurement as independent variables (e.g., TBR at follow-up = intervention + TBR at baseline). We used linear regression models because analyzing change scores does not appropriately control baseline imbalances (20). Because baseline CT calcification mass, phylloquinone intake, and a low ABI differed between the intervention and placebo groups at baseline, we adjusted for these variables in a secondary analysis. Due to the baseline difference in calcification mass between treatment arms and the strong predictive value of baseline calcification mass on progression of calcification mass (21, 22), we assessed the correlation between baseline and difference between baseline and follow-up measurements for calcification mass and TBR using Spearman's correlation coefficients. Because baseline calcification mass was correlated with change in calcification mass, we excluded participants with no calcification at baseline and with a calcification mass score >1000 (arbitrarily chosen) in post-hoc analyses.

All measurements and analyses were performed blinded to treatment. There were no missing data for the imaging, the outcome measurement of interest. In the adjusted models, 2 participants had missing information on phylloquinone intake, and median phylloquinone intake was imputed. Analyses were performed with R version 3.2.2, and *P* < 0.05 was considered statistically significant.

## Results

**FIGURE 1:**
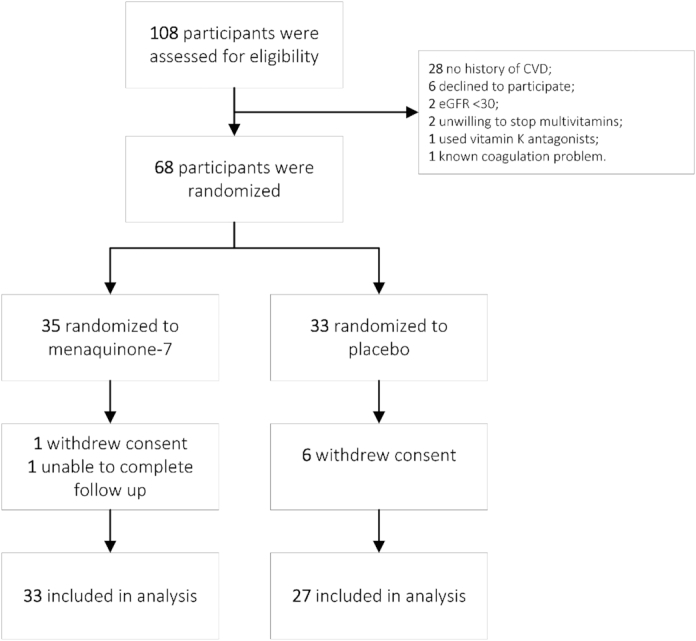
Flowchart of study participation. CVD, cardiovascular disease; eGFR, estimated glomerular filtration rate.

A total of 108 patients were screened for participation in this study; 40 patients were ineligible, mainly because of the absence of CVD or because they declined to participate (**Figure 1**). Sixty-eight patients were randomly assigned: 35 to the MK-7 group and 33 to the placebo group. Eight patients were lost to follow-up (MK-7 group: 2; placebo group: 6). Reasons for loss to follow-up were unrelated to the intervention and mainly due to technical problems during the first PET/CT acquisition.

Baseline characteristics are shown in **Table 1**. The study included 16 women (24%), and the mean age was 69.1 y (SD = 8.4). Participants in the MK-7 group had higher baseline calcification levels, higher phylloquinone intake, and more often a low ABI compared with the placebo group. Baseline characteristics of participants with a complete follow-up were comparable to those of the randomly assigned participants (**Supplemental Table 1**).

**TABLE 1:** Baseline characteristics of all randomly assigned participants

Characteristics	Vitamin K (*n* = 35)^1^	Placebo (*n* = 33)^1^
Age, y	69.1 ± 8.4	69.1 ± 8.4
Women, *n*	9 (25.7)	7 (21.2)
BMI, kg/m²	31.1 ± 5.6	31.1 ± 5.0
Systolic BP, mm Hg	136.0 ± 20.5	137.8 ± 14.3
Diastolic BP, mm Hg	69.8 ± 11.4	73.5 ± 9.5
Current smoker, *n*	6 (17.1)	4 (12.1)
Higher educated, *n*	15 (42.9)	13 (39.4)
Vitamin D supplements, *n*	23 (65.7)	24 (72.7)
ABI ≤ 0.9	16 (45.7)	9 (27.3)
Laboratory measurements		
HbA1c, mmol/mol	57.1 ± 14.8	59.6 ± 17.1
eGFR, mL/min/1.73 m²	79.2 ± 26.2	86.6 ± 26.1
Total cholesterol, mmol/L	4.5 ± 1.3	4.2 ± 1.2
HDL cholesterol, mmol/L	1.1 ± 0.3	1.1 ± 0.3
LDL cholesterol, mmol/L	2.1 ± 0.9	2.0 ± 0.9
Triglycerides, mmol/L	2.8 (1.8–3.4)	1.9 (1.5–2.7)
dp-ucMGP, pmol/L	613 (513–684)	615 (489–743)
Energy-adjusted vitamin K intake		
Total vitamin K, microgram	167 (138–288)	141 (117–193)
Phylloquinone, mg	124.8 (100–225)	94 (73–149)
Menaquinones, mg	49 (35–57)	48 (33–52)
Calcification measurements		
TBR	2.2 ± 0.7	2.1 ± 0.6
CT calcification mass	196.0 (32.5–424.0)	44.9 (9.6–409.5)

1 Values are means ± SDs, medians (IQRs), or *n* (%). ABI, ankle–brachial index; BP, blood pressure; CT, computed tomography; dp-ucMGP, dephosphorylated–uncarboxylated matrix Gla protein; eGFR, estimated glomerular filtration rate; HbA1c, glycated hemoglobin; TBR, target-to-background ratio.

**FIGURE 2:**
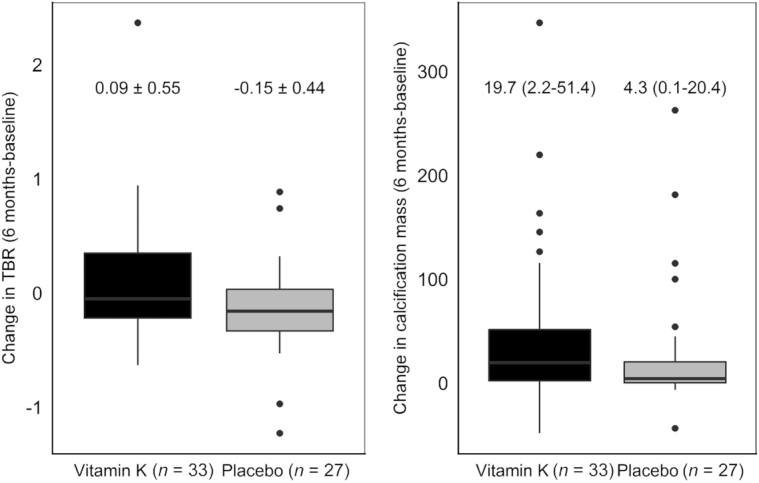
Absolute changes in TBR (left) and calcification mass (CT; right) in the placebo and vitamin K group between baseline and 6-mo intervention, presented as medians ± SDs and medians (IQRs), respectively. CT, computed tomography; TBR, target-to-background ratio.

The absolute differences between baseline and 6-mo intervention are presented in **Figure 2**. After 6-mo intervention, TBR tended to increase, with 0.25 in the MK-7 group (95% CI: –0.02, 0.51; *P* = 0.06) compared with placebo. Log-transformed calcification mass did not increase in the MK-7 group compared with placebo (0.50; 95% CI: −0.24, 1.23; *P* = 0.18), although this result was not statistically significant (**Table 2**). Adjustment for baseline characteristics (baseline calcification mass, phylloquinone intake, and low ABI) did not alter these results.

**TABLE 2:** Results of linear regression analyses and sensitivity analyses excluding participants without baseline calcification and with high baseline calcification levels

	Linear regression	*P* value
TBR (*n* = 60)		
Model 1	0.25 (−0.02, 0.51)	0.06
Model 2	0.31 (0.02, 0.60)	0.03
Sensitivity analyses 1 (*n* = 57)		
Model 1	0.24 (−0.03, 0.52)	0.08
Model 2	0.32 (0.02, 0.62)	0.04
Sensitivity analyses 2 (*n* = 54)		
Model 1	0.24 (−0.04, 0.52)	0.09
Model 2	0.34 (0.04, 0.64)	0.03
Calcification mass (*n* = 60)^1^		
Model 1	0.50 (−0.24, 1.23)	0.18
Model 2	0.40 (−0.36, 1.16)	0.30
Sensitivity analyses 1 (*n* = 57)^1^		
Model 1	0.22 (0.04, 0.41)	0.02
Model 2	0.27 (0.08, 0.47)	<0.01
Sensitivity analyses 2 (*n* = 54)^1^		
Model 1	0.42 (−0.32, 1.16)	0.26
Model 2	0.41 (−0.37, 1.19)	0.29

1 Values are log-transformed values (95% CIs). Model 1: linear regression models included outcome measurement as the dependent variable, and intervention and baseline measures were used as independent values. Model 2: additionally adjusted for calcification mass, phylloquinone intake, and ankle–brachial index. Sensitivity analyses 1: exclusion of 3 participants with no baseline calcification mass (CT), *n* = 57. Sensitivity analyses 2: exclusion of 6 participants with baseline calcification mass score <1000 (CT), *n* = 54. CT, computed tomography; TBR, target-to-background ratio.

TBR and calcification mass were modestly correlated at baseline (*r* = 0.47; 95% CI: 0.27, 0.64). Furthermore, baseline calcification mass was modestly correlated with change in calcification mass between baseline and 6 mo (*r* = 0.53; 95% CI: 0.32, 0.69), whereas TBR at baseline was not correlated with change in TBR levels during follow-up (*r* = −0.01; 95% CI: −0.35, 0.15). Finally, change in TBR was not correlated with change in calcification mass (*r* = 0.14; 95% CI: −0.12, 0.38). Based on the correlation between calcification at baseline and change in calcification mass, we excluded participants with no calcification at baseline (*n* = 3), which resulted in a similar effect of MK-7 compared with placebo on TBR for 6-mo intervention (Table 2), whereas calcification mass significantly increased (model 1: 0.22; 95% CI: 0.04, 0.41; *P* = 0.02). When excluding participants with a high calcification mass at baseline (*n* = 3), the results were similar to those in the full study population for both TBR and calcification mass.

**FIGURE 3:**
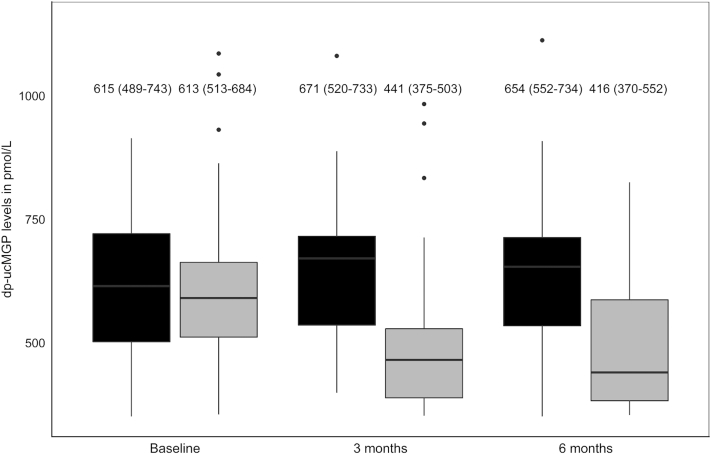
dp-ucMGP concentrations in the placebo and vitamin K group at baseline, 3 mo, and 6 mo intervention, including medians and IQRs. The gray boxes represent the vitamin K group (*n* = 33), and the black boxes represent the placebo group (*n* = 27). dp-ucMGP, dephosphorylated–uncarboxylated matrix Gla protein.

MK-7 supplementation significantly reduced inactive MGP concentrations after 3 mo of intervention compared with placebo (−205.6 pmol/L; 95% CI: −255.8, −155.3 pmol/L; *P* < 0.01) (**Figure 3**). This effect of MK-7 compared with placebo was sustained after 6 mo (−202.7 pmol/L; 95% CI: −272.5, −132.8 pmol/L; *P* < 0.01), indicating high compliance. According to pill count, compliance was also high: 97.4% (95% CI: 92.3%, 99.1%) in the intervention group and 97.8% (95% CI: 94.2%, 99.7%) in the placebo group. No serious adverse events were reported.

## Discussion

In contrast to our hypothesis, active vascular calcification on ^18^F-NaF PET scan tended to increase after MK-7 supplementation compared with placebo during 6-mo intervention. In addition, no effect of MK-7 supplementation on CT calcification mass was found. Therefore, this study does not support that MK-7 supplementation inhibits vascular calcification.

This study has several strengths. First, it is a randomized, double-blind, placebo-controlled design with high compliance and interobserver reliability for assessment of imaging. Second, this is the first study to investigate the effect of MK-7 supplementation in patients with type 2 diabetes, who are known to be prone to medial calcification (23). Finally, an innovative measurement of vascular calcification was used as the primary endpoint, allowing sensitive detection of changes in vascular calcification activity (13). However, some limitations need to be addressed, which might partially explain the unexpected results. The dropout rate was relatively high, especially in the placebo group. Although the reasons for dropout were unrelated to treatment, it may have reduced power to detect the effects of vitamin K supplementation. However, if we recalculate the sample size using the mean and SD of this study, the study has the statistical power of 85% to detect changes in vascular calcification despite the high dropout rate. Importantly, the baseline characteristics of the subjects who completed follow-up were comparable to those of all randomly assigned participants, making selection bias unlikely. However, the primary outcome parameter—that is, vascular calcification—was unexpectedly higher in the vitamin K treatment group, despite randomization, which might have influenced the results. Finally, our diabetes patient cohort did not show a severe vitamin K deficiency.

A previous study investigating the effect of phylloquinone supplementation on coronary artery calcification showed a protective effect of vitamin K supplementation in older men and women (8). In addition, a recent study in patients with aortic valve calcification supplemented with vitamin K for 1 y found a 50% inhibition of progression of vascular calcification (9). Our study is the first to assess the effect of MK-7 on vascular calcification measured by ^18^F-NaF activity. To date, the effect of MK-7 has been studied only in association with arterial stiffness, which showed a beneficial effect of MK-7 supplementation on pulse wave velocity (10, 11). Only 1 study did not detect an effect of MK-7 supplementation on arterial stiffness and physical function during 6-mo follow-up, which was probably due to the low-dose MK-7 supplementation (100 µg) and relative short follow-up period to detect changes in arterial stiffness (12). The MK-7 dosage in our study was much higher than that in the previously mentioned trial. The follow-up period in our study was similar to that of the previous study, but because ^18^F-NaF PET scan is designed to detect early changes in calcification, it is not likely that the follow-up period explains our results.

Based on the previously mentioned studies, we hypothesized that MK-7 supplementation would hold vascular calcification progression, resulting in lower ^18^F-NaF PET activity. Contrary to our hypothesis, ^18^F-NaF activity tended to increase in the MK-7 group compared with the placebo group, although these results lacked statistical significance. One reason for this unexpected result might be the difference in calcification mass at baseline, which was higher in the MK-7 group than in the placebo group, despite randomization. Previous studies on determinants of coronary artery calcification (CAC) progression showed that a high baseline CAC burden was the strongest predictor of CAC progression (21, 22). In line with this, in our study a high baseline calcification mass was correlated with an increase in calcification mass during follow-up. Because calcification mass was higher in the MK-7 group at baseline, this may explain the higher progression of vascular calcification in this group compared with the placebo group. However, excluding participants with high baseline calcification mass only slightly attenuated the effect of MK-7 supplementation on change in calcification mass compared with placebo and did not alter our results for TBR.

Another explanation for these results may concern the interpretation of ^18^F-NaF PET activity as a marker of active calcification. ^18^F-NaF PET activity is promising to detect early changes in active deposition of vascular calcification probably before calcifications become visible on CT, due to the binding of the tracer to hydroxyapatite, thereby enabling the detection of microcalcifications (24). Our study is the second trial using ^18^F-NaF PET activity for treatment follow-up. One previous trial investigating the effect of etidronate on vascular calcification in patients with pseudoxanthoma elasticum found no effect on ^18^F-NaF PET activity in the femoral artery, whereas CT images showed a significant reduction of vascular calcification in the intervention group (25). This challenges our understanding of what ^18^F-NaF PET activity in femoral arteries actually represents. In addition, in some studies, some areas of macrocalcification showed no ^18^F-NaF uptake, whereas other areas with microcalcification showed high ^18^F-NaF uptake (26). Macrocalcifications have a larger volume but a small surface area, whereas microcalcifications have a large surface area. Because ^18^F-NaF is only incorporated into the outer surface, our results might reveal a shift from macro- to microcalcification with MK-7 supplementation, by which ^18^F-NaF uptake increases as seen in our results (26). In our study, changes in ^18^F-NaF PET activity were directionally concordant with changes in calcification mass; therefore, this does not fully explain our results. Currently, multiple studies are investigating whether ^18^F-NaF PET activity can identify the vulnerable atherosclerotic plaque, which might shed light on the interpretation of our study results.

Finally, our hypothesis could be influenced by the absence of a severe vitamin K deficiency in patients with type 2 diabetes, although the dp-ucMGP concentrations in this study showed some evidence of low vitamin K concentrations because dp-ucMGP >300 pmol/L is considered to be in the normal healthy range. Vitamin K deficiency has been shown to be a major contributor to vascular calcification in other patients groups with more severe deficiencies, such as chronic kidney disease patients. Results of ongoing clinical trials studying the effect of MK-7 on vascular calcification in these patient populations are awaited.

In conclusion, vitamin K supplementation tended to increase ^18^F-NaF activity compared with placebo and did not hold or reduce active calcification. In addition, the use of vitamin K supplementation did not reduce progression of vascular calcification as measured by conventional CT. Results of ongoing trials with vitamin K supplementation and further research investigating the interpretation of ^18^F-NaF PET activity coupled with higher-resolution imaging are needed to fully understand the effects of vitamin K on vascular calcification.

## Supplementary Material

nqz147_Supplemental_File

## Notes

JWJB and SRZ are supported by the Senior Dr. Dekker grant (2013T120) from the Dutch Heart Foundation.

Data sharing: Data described in the article, code book, and analytic code will be made available upon request.

Supplemental Table 1 is available from the “Supplementary data” link in the online posting of the article and from the same link in the online table of contents at https://academic.oup.com/ajcn/.

Abbreviations used: ABI, ankle–brachial index; CAC, coronary artery calcification; CT, computed tomography; CVD, cardiovascular disease; dp-ucMGP, dephosphorylated–uncarboxylated matrix Gla protein; eGFR, estimated glomerular filtration rate; MGP, matrix Gla protein; MK-7, menaquinone-7; PET, positron emission tomography; SUV_max_, maximal standardized uptake value; SUV_mean_, mean standardized uptake value; TBR, target-to-background ratio; ^18^F-NaF PET, ^18^sodium fluoride positron emission tomography.
