# Supplementary material for: The effect of menaquinone-7 supplementation on vascular calcification in patients with diabetes: a randomized, double-blind, placebo-controlled trial
Source: Am J Clin Nutr. 2019 Aug 6;110(4):883–90. doi: 10.1093/ajcn/nqz147 (PMC6766434; doi:10.1093/ajcn/nqz147)
Supplement: nqz147_Supplemental_File [file nqz147_supplemental_file.docx]

| **Supplementary Table 1.** Baseline characteristics of all participants with complete follow up stratified by treatment. | | |
| --- | --- | --- |
|  | **Vitamin K (n=33)** | **Placebo (n=27)** |
| Age _(years)_ | 69.8 ± 8.1 | 69.2 ± 7.2 |
| Females | 7(21.2) | 7 (25.9) |
| BMI _(kg/ m²)_ | 31.0 ± 5.7 | 31.4 ± 5.2 |
| Systolic BP _(mmHg)_ | 136.4 ± 20.6 | 136.5 ± 14.7 |
| Diastolic BP _(mmHg)_ | 69.2 ± 11.5 | 73.3 ± 9.6 |
| Current smoker | 4 (12.1) | 3 (11.1) |
| Higher educated | 14 (42.5) | 11 (40.7) |
| Vitamin D supplements | 21 (63.6) | 20 (74.1) |
| ABI ≤ 0.9 | 15 (45.5) | 6 (22.2) |
| *Laboratory measurements* | | |
| HbA1c _(mmol/mol)_ | 57.1 ± 14.8 | 59.6 ± 17.1 |
| eGFR _(ml/min/1.73m²)_ | 80.7 ± 26.2 | 87.0 ± 26.4 |
| Total cholesterol _(mmol/L)_ | 4.5 ± 1.3 | 4.2 ± 1.2 |
| HDL cholesterol _(mmol/L)_ | 1.1 ± 0.3 | 1.1 ± 0.3 |
| LDL cholesterol _(mmol/L)_ | 2.1 ± 0.9 | 2.0 ± 0.9 |
| Triglycerides _(mmol/L)_ | 2.8 (1.8-3.4) | 1.9 (1.5-3.2) |
| Dp-ucMGP _(pmol/L)_ | 608 (512-673) | 633 (530-768) |
| *Energy adjusted vitamin K intake* | | |
| Total vitamin K _(mg)_ | 171 (140-293) | 134 (115-183) |
| Phylloquinone _(mg)_ | 126 (101-232) | 82 (67-131) |
| Menaquinones _(mg)_ | 51 (37-57) | 44 (33-55) |
| *Calcification measurements* | | |
| TBR | 2.2 ± 0.7 | 2.1 ± 0.6 |
| CT calcification mass | 215.9 (37.6-447.3) | 44.9 (10.1-442.5) |
| Values are mean ± SD, median (IQR) or n (%).  BMI: Body Mass Index, HbA1c: Hemoglobin A1c, eGFR: estimated Glomerular Filtration Rate, HDL: high density lipoprotein, LDL: low density lipoprotein, dp-ucMGP: dephosphorylated-uncarboxylated Matrix Gla Protein, CT: Computed Tomography, TBR: Target to Background Ratio | | |

**Supplementary Material**
